# Evolution and Antibacterial Evaluation of 8-Hydroxy-cycloberberine Derivatives as a Novel Family of Antibacterial Agents Against MRSA

**DOI:** 10.3390/molecules24050984

**Published:** 2019-03-11

**Authors:** Yuan-Shuai Yang, Wei Wei, Xin-Xin Hu, Sheng Tang, Jing Pang, Xue-Fu You, Tian-Yun Fan, Yan-Xiang Wang, Dan-Qing Song

**Affiliations:** Beijing Key Laboratory of Antimicrobial Agents, Institute of Medicinal Biotechnology, Chinese Academy of Medical Sciences and Peking Union Medical College, Beijing 100050, China; yangmarshal88@163.com (Y.-S.Y.); weiwei082695@163.com (W.W.); huxinxin1985@163.com (X.-X.H.); tang13874204108@163.com (S.T.); pangjing.pangjing@163.com (J.P.); 13311123098@163.com (X.-F.Y.); songdanqingsdq@hotmail.com (D.-Q.S.)

**Keywords:** cycloberberine, anti-MRSA, structure–activity relationship, topoisomerase IV

## Abstract

Twenty-five new derivatives of 8-hydroxycycloberberine (**1**) were synthesized and evaluated for their activities against Gram-positive bacteria, taking **1** as the lead. Part of them displayed satisfactory antibacterial activities against methicillin-susceptible *Staphylococcus aureus* (MSSA) and methicillin-resistant *Staphylococcus aureus* (MRSA), as well as vancomycin-intermediate *Staphylococcus aureus* (VISA). Especially, compound **15a** displayed an excellent anti-MRSA activity with MICs (minimum inhibitory concentrations) of 0.25–0.5 μg/mL, better than that of **1**. It also displayed high stability in liver microsomes and whole blood, and the LD_50_ value of over 65.6 mg·kg^−1^ in mice via intravenous route, suggesting a good druglike feature. The mode of action showed that **15a** could effectively suppress topo IV-mediated decatenation activity at the concentration of 7.5 μg/mL, through binding a different active pocket of bacterial topo IV from quinolones. Taken together, the derivatives of **1** constituted a promising kind of anti-MRSA agents with a unique chemical scaffold and a specific biological mechanism, and compound **15a** has been chosen for the next investigation.

## 1. Introduction

Methicillin-resistant *Staphylococcus aureus* (MRSA), classified as serious threat pathogen, has become a main cause of hospital and community-acquired infections across the world [1,2,3,4]. In the US alone, at least two million illnesses and 23,000 deaths are caused by multidrug-resistant bacterial infections every year according to data released by the Centers for Disease Control and Prevention (CDC) [1]. Thereinto, around 19,000 deaths and 360,000 hospitalizations resulted from infections of MRSA along with $3–4 billion in healthcare costs [5]. Vancomycin, linezolid and daptomycin have been used as the last resort for MRSA infections in the clinic. Unfortunately, vancomycin-intermediate *S. aureus* (VISA), heteroresistant VISA (hVISA), vancomycin-resistant *S. aureus* (VRSA), linezolid-resistant *S. aureus* and daptomycin-resistant *S. aureus* have been successively reported [6,7,8,9,10]. Especially, MRSA/VISA have been listed as global priority pathogens of antibiotic-resistant bacteria released by the World Health Organization (WHO) on Feb 27th 2017 [11], and the treatment of MRSA/VISA infections has become severe concerns across the world. Therefore, great efforts must be made to explore alternative agents witH-Novel structure scaffold or mechanism of action for the treatment of invasive life-threatening infections arising from MRSA/VISA.

**Figure 1:**
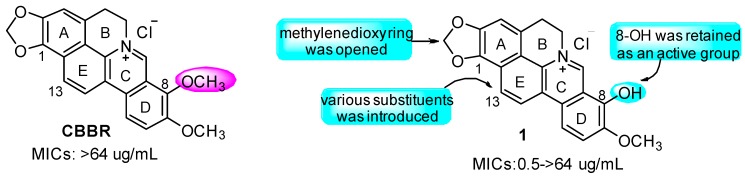
Structures of CBBR and **1** as well as modification strategies.

In the past several years, our team has been committed to the discovery and development of novel antibacterial agents against multidrug-resistant pathogens, witH-Novel chemical entities and biological mode of action [12,13]. We first discovered that the cycloberberine (CBBR, Figure 1), generated from berberine (BBR) in our lab [14], was a special molecular scaffold against both methicillin-susceptible *S. aureus* (MSSA) and MRSA, as well as VISA [15,16]. The primary structure–activity relationships (SAR) demonstrated that introducing a suitable mono-substituent at the 8- or 13-position of CBBR could significantly enhance the antibacterial activity against MSSA/MRSA [15,16], though CBBR itself had no bactericidal activity at all, as shown in Figure 1. The top compound 8-hydroxycycloberberine (**1**, Figure 1) displayed a promising effect against the tested strains such as MRSA with MIC values of 0.5−>64 μg/mL, which is much higher than that of levofloxacin (Lev) widely used in the clinic.

Interestingly, compared with CBBR possessing a 8-OCH_3_ group, compound **1** with 8-OH showed a greatly significant improvement in activity against both MSSA and MRSA, indicating that the 8-OH function might play a critical role in enhancing the antibacterial effects. The unique chemical scaffold and biological activity of **1** encouraged us to continue a new round of SAR of its derivatives, aiming at developing these compounds into a new class of drug candidates against MRSA. Therefore, in the present study, as depicted in Figure 1, taking compound **1** as the lead, the 8-OH in CBBR was retained, and various substituents were respectively attached on the 13-position, by which a series of new **1** derivatives were designed, synthesized and then evaluated for their antibacterial effect against MSSA and MRSA. Additionally, the stability in liver microsomes and whole blood, toxicity in vivo as well as the bactericidal mechanism studies of the representative compounds were carried out as well.

## 2. Results and Discussion

### 2.1. Chemistry

**Scheme 1:**
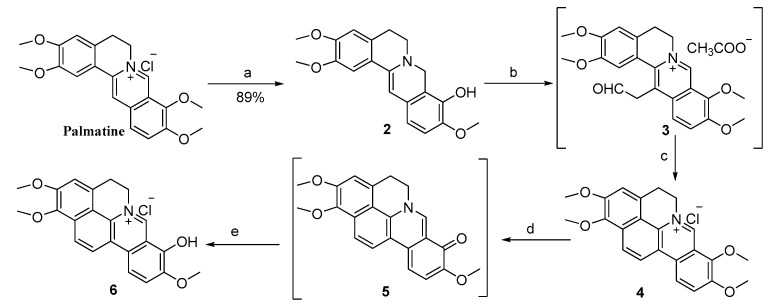
Reagents and conditions: (**a**) NaBH_4_, 5% NaOH/K_2_CO_3_, CH_3_OH, room temperature, 3 h; (**b**) 40% glyoxal, HOAc/CH_3_CN, reflux, 6 h; (**c**) CH_3_OH/HCl (2/1 by vol.), room temperature, 24 h, 62% (over two steps); (**d**) 20–30 mmHg, 195–210 °C, 40 min; (**e**) HCl/EtOH (5/95 by vol.), 90% (over two steps).

**Scheme 2:**
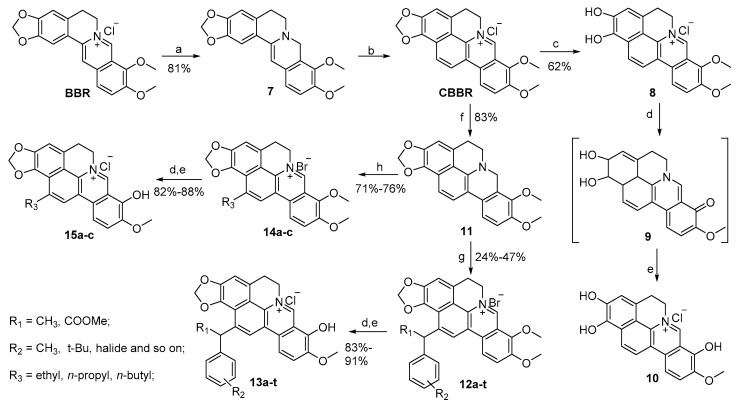
Reagents and conditions: (**a**) NaBH_4_, 5% NaOH, K_2_CO_3_, CH_3_OH, r.t., 3 h; (**b**) (i) 40% glyoxal, HOAc, CH_3_CN, reflux, 6 h; (ii) CH_3_OH/HCl (2/1 by vol.), room temperature, 24 h, 67% (over two steps); (**c**) benzene-1,3,5-triol, 60% H_2_SO_4_; (**d**) 20–30 mmHg, 195–210 ^o^C, 40 min; (**e**) HCl/EtOH (5/95 by vol.), 39% (over two steps); (**f**) NaBH_4_, K_2_CO_3_, CH_3_OH, room temperature, 12 h; (**g**) substituted benzyl bromide, K_2_CO_3_ (2.0 eq.), CH_3_CN, reflux, 3–24 h; (**h**) aliphatic aldehyde, HOAc, 80% EtOH, reflux, 8 h.

The synthetic routes to all twenty-five target compounds are displayed in Scheme 1 and Scheme 2 respectively. As shown in Scheme 1, palmatine was selectively reduced to dihydropalmatine (**2**) in 89% yield using NaBH_4_ as a reducing agent in the presence of 5% NaOH/K_2_CO_3_ using a previous procedure [14]. Compound **2** was then reacted with 40% glyoxal in refluxing HOAc/CH_3_CN to give the intermediate 13-acetaldehyde palmatine (**3**), to which CH_3_OH/HCl (2/1 by vol.) was added directly to complete the cyclization reaction and the desired product cyclopalmatine (**4**) was obtained in an overall yield of 62%. Then, compound **4** was heated at 195–210 °C under vacuum (20–30 mmHg) to get the key intermediate **5**. Finally, product **6** was acquired with a combined 90% conversion via acidification of compound **5** with concentrated HCl/EtOH (5/95 by vol.) by a classic keto-enol tautomerism.

As described in Scheme 2, CBBR was prepared from BBR using a previously reported procedure [14]. Compound **8** was prepared via demethylenation of CBBR with benzene-1,3,5-triol and 60% H_2_SO_4_ in a yield of 62%. The demethylation of **8** yielded intermediate **9**, which was converted quickly to 1,2,8-trihydroxyl **10** in the presence of concentrated HCl/EtOH (5/95) in a combined 39% yield. Dihydrocycloberberine (**11**) was prepared in 83% yield via reduction of CBBR witH-NaBH_4_ as a reducing agent in CH_3_OH in the presence of K_2_CO_3_ [15]. The compounds in series **12** were generated in 24–47% yields from **11** and diverse substituted benzyl bromides at reflux temperature. 

Similarly, compounds **12a**–**t** were heated at 195–210 °C for 40 min under vacuum (20–30 mmHg) and acidified using concentrated HCl/EtOH (5/95) to produce the target compounds **13a**–**t** in good yields. Meanwhile, the products in series **14** were obtained in good yields from **11** and diverse aliphatic aldehydes in HOAc and 80% EtOH at reflux temperature. The products **15a**–**c** were generated from **14a**–**c** using a similar synthetic method as for compounds **13a**–**t**. All the desired products were purified with flash column chromatography on silica gel using CH_2_Cl_2_ and CH_3_OH as eluents or washed with 95% EtOH. The ^1^H-NMR, ^13^C-NMR and HRMS data of compound **15a** is provided in Supplementary Materials. 

### 2.2. Pharmacological Evaluation

#### 2.2.1. SAR for Anti-Bacterial Activity

SAR study for antibacterial activity against drug-susceptible Gram-positive bacteria was initially carried out, taking Lev and compound **1** as reference drugs. The chemical structures of 25 target compounds and their MIC values against a panel of drug-susceptible strains [17], including MSSA, methicillin-susceptible *S. epidermidis* (MSSE), *S. saprophyticus* (*S. s*) and *S. hominis* (*S. h*), were summarized in Table 1. Bacteria strains used in this study were from the ATCC collection and clinical isolates in Chinese hospitals. The influence of the methylenedioxy ring was explored first, and compounds **6** and **10** were created and tested. As shown in Table 1, both of them lost the antimicrobial activities partially or completely, suggesting that methylenedioxy ring might be essential for the activity against Gram-positive strains.

Then, we moved SAR analysis to the influence of substituent at the 13-position in compound **1**. Our previous SAR results on CBBR indicated that the introduction of a 13-benzyl motif was beneficial for the activity [16], therefore, various benzyl moieties with electron-donating and electron-withdrawing groups on the benzene ring were respectively introduced into position 13, and 20 new analogs **13a–t** were constructed and tested. As described in Table 1, compound **13a** with a 13-benzyl displayed moderate antimicrobial activity with MICs of 8–16 μg/mL, while the compounds **13b**–**d** with fluoro atoms as an isostere of the hydrogen atom lost their potency to varying degrees. However, the compounds **13e** and **13f** with *p*-chloro or *p*-bromo groups showed preferable antimicrobial activities with MIC values of 2–8 μg/mL. Compounds **13h** and **13i** bearing *m*-methyl and *p*-methyl gave potent activities with MICs of 2–16 μg/mL, less than that of lead **1**. Next, compounds **13o** and **13p** with *p*-CN and *o*-NO_2_ respectively displayed moderate antimicrobial activities with most MIC values in the 4–16 μg/mL range, while other compounds with electron-withdrawing groups showed partially or completely diminished antimicrobial activities. 

Furthermore, a methyl or methyloxycarbonyl group was introduced to the *α*-C atom of ring E to generate **13s** and **13t**, respectively, and both of them showed decreased activity to different degrees. Finally, an ethyl, *n*-propyl or *n*-butyl were respectively introduced at the 13-position of **1**, whereby three new analogs **15a**–**c** were prepared and investigated. All of them exhibited good activities against the tested strains with MIC values of 0.25–16 μg/mL, and compound **15a** showed especially potent activities, with MIC values of 0.25–1.0 μg/mL, superior or similar to those of the lead compound **1**. The results thus indicate that introducing a small-sized substituent at the 13-position might enhance the activity.

All target compounds were then measured for their antibacterial activities against drug-resistant organisms, such as MRSA/VISA and methicillin-resistant *S. epidermidis* strains (MRSE). As listed in Table 2, their potencies against MRSE/MRSA/VISA strains were basically consistent with those against drug-susceptible ones. Most of compounds showed potential anti-MRSA and anti-VISA effects with MIC values ranging from 1 to 64 μg/mL. Compounds **13e**–**f**, **13h**–**i**, **13o**–**p** and **15a**–**c** displayed promising effects, with MIC values ranging from 0.25 to 8 μg/mL. Especially, compound **15a** displayed an excellent effect against MRSA and VISA with MIC values of 0.25–0.5 μg/mL, better than that of **1**, and then it was selected as the representative compound for the next investigation.

#### 2.2.2. Time-kill Curve Study of **15a**

**Figure 2:**
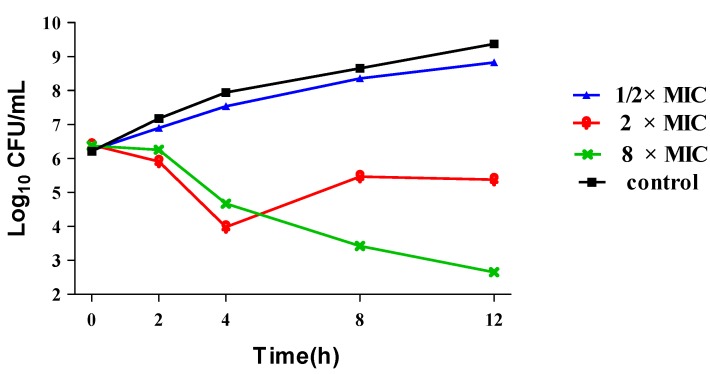
Time-kill curve assay of compound **15a** over a 12 h incubation period at 37 °C against MRSA 18-4 (MIC = 0.5 μg/mL). Samples were taken at 2, 4, 8 and 12 h to determine viable bacterial numbers.

To further confirm the antibacterial activities of this kind of compounds, the time–kill curve assay of compound **15a** for MRSA strain isolated from Chinese patients was carried out subsequently with DMSO as a negative control. As shown in Figure 2, distinct reductions of the viable cell counts were observed after 4 h exposure of the bacteria to **15a** at concentrations of 2× and 8×MIC. Especially, it exhibited significant time-dependent action and the maximum reduction in viable counts was over 6 log_10_CFU/mL in 12 h for the 8× MIC, therefore, it was determined that **15a** was a marginal bactericide [18] at the concentration of 8× MIC.

#### 2.2.3. Evaluation of Metabolic Stability and Acute Toxicity Assay of Compound **15a**

The metabolic stability of compound **15a** in liver microsomes was conducted in human and rat as well as mouse with the clinical reagent testosterone (Tes) as reference. As depicted in Table 3, compared to Tes, compound **15a** showed an obviously longer half-life (T_1/2_) and residual ratio, as well as lower microsomal intrinsic clearance in all three species. Especially, the T_1/2_ of compound **15a** was over 105 min and the remaining ratio was still 70.6% after 60 min in human liver microsomes, indicating a good characteristic of metabolic stability.

Next, a stability assay of compound **15a** was carried out in whole blood in vitro, taking enalapril (Ems) [15,16] as the positive control. As indicated in Table 4, as expected, compound **15a** gave an exciting stability profile and the remaining ratio was still over 85.4% after 24 h in blood, while Ems was completely decomposed within three hours. The results suggest that **15a** might possess an ideal in vivo metabolic stability.

Then, acute toxicity test on compound **15a** was performed in Kunming mice through intravenous route. The results indicated that compound **15a** gave a half-lethal dose (LD_50_) value of 65.6 mg·kg^−1^, suggesting a good safety profile.

#### 2.2.4. Preliminary Mechanism of Compound **15a**

**Figure 3:**
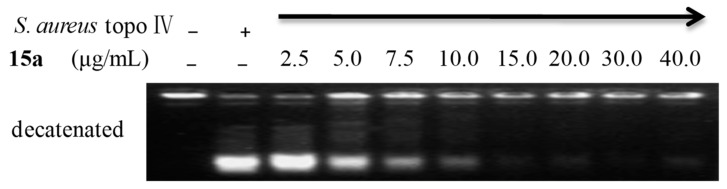
Inhibition of *S. aureus* topo IV. In all panels, enzyme and **15a** are absent (-) in lane 1, enzyme present (+) and **15a** absent in lane 2, and enzyme (arrow) and **15a** present in lanes 3–10 with different concentration. Decatenation activity of topo IV was partially blocked by **15a** (lanes 4, 5, 6) and completely blocked by **15a** (lanes 7, 8, 9, 10).

Taking into account the planar rigid structure similarity between **15a** and quinolones, we initially examined its effects on gyrase and topoisomerase (topo) IV activities of *S. aureus* by gyrase-mediated supercoiling of relaxed DNA assay and topo IV-mediated decatenation of kinetoplast DNA (kDNA) assay [19,20], respectively. As shown in Figure 3, topo IV-mediated decatenation activity was significantly inhibited in a concentration-dependent manner after treatment of **15a**. It could partially inhibit topo IV activity at the concentration of 7.5 μg/mL (lane 5), and block almost completely the decatenation reaction at the concentration of 15 μg/mL (lane 7). However, inhibition activity on gyrase-mediated supercoiling was not distinctly observed after treatment with **15a** at even upped to 100 μg/mL. These results indicated that **15a** could effectively suppress the topo IV-mediated decatenation activity, but a different mode of action from quinolones. 

#### 2.2.5. Molecular Docking Study of Key Compound **15a**

**Figure 4:**
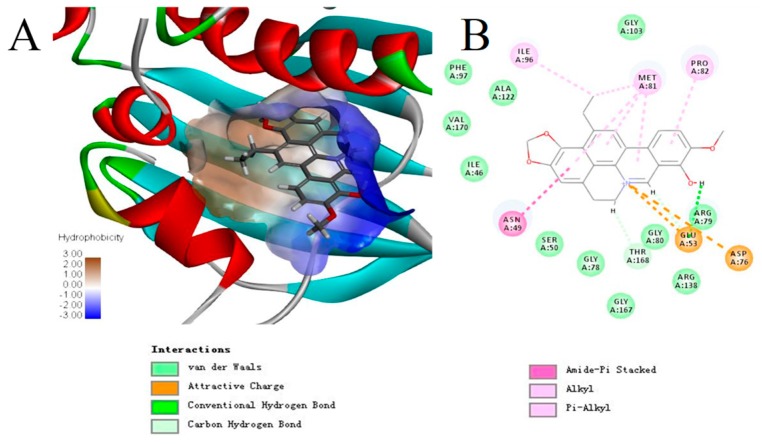
(**A**) Solid surface map of the interaction pocket with compound **15a**. Red, blue, and white colored regions correspond to negatively charged, positively charged, and neutral areas, respectively. (**B**) Binding modes within the receptor *S. aureus* topo IV active pocket. These figures were produced using the Discovery Studio 4.5 software. The receptor structure is shown in surface form. Key bonds are indicated by dashed lines between the atoms involved, and the colors of key bonds and residues are shown according to the interaction mode (van der Waals, light green; attractive charge, orange; hydrogen bond, green; carbon hydrogen bond, celadon; amide-π stacked, amaranth; alkyl and π-alkyl, mauve pink).

It was reported that topo IV enzyme of Gram-positive bacteria possesses at least two active pockets, one is the well-known quinolone-binding cavity and the other is kibdelomycin-binding site [21,22,23]. In view of the good activity of compound **15a** against quinolone-resistant MRSA or VISA, we performed molecular docking between the topo IV binding site of kibdelomycin and compound **15a** interactions using Discovery Studio 4.5 software (Edition 4.5; BIOVIA: San Diego, CA, USA, 2015) [17,24]. As expected, compound **15a** fit well in the active hydrophobic pocket of the binding site (Figure 4A, brown area). One conventional hydrogen bond with GLU53, two attractive charges formed by nitrogen ions with ASP76 and GLU53, van der Waals forces and hydrophobic interactions (Figure 4B) contributed together to the strong interactions. The results suggested that **15a** might bind to the kibdelomycin-pocket site on topo IV, a different mode of action from the quinolones widely used in the clinic. 

## 3. Experimental Section

### 3.1. Apparatus, Materials, and Analysis Reagents

Melting points (m.p.) were obtained with a CXM-300 melting point apparatus (Shanghai Changfang Optical Instrument Co., Ltd., Shanghai, China) and are uncorrected. The ^1^H-NMR spectra was recorded on an Inova 500 or 600 MHz spectrometer (Varian, San Francisco, CA, USA) and ^13^C-NMR on an Avance III 400, 500 or 600 spectrometer (Bruker, Zürich, Switzerland), with Me_4_Si as the internal standard. High-resolution mass spectra (HRMS-ESI) data was recorded on an Autospec UItima-TOF mass spectrometer (Micromass UK Ltd., Manchester, UK). Flash chromatography (particle size 0.038 mm) was performed on a CombiflashRf 200 system (Teledyne, Lincoln, NE, USA). 

### 3.2. Chemistry

#### 3.2.1. Synthesis of 1,2-Dimethoxy-8-hydroxy-9-methoxycycloberberine Chloride (**6**)

Palmatine (7.7 g, 20 mmol) and K_2_CO_3_ (8.3 g, 60 mmol) were dissolved in CH_3_OH (250 mL), and then 5% NaOH (10 mL) solution containing NaBH_4_ (0.83 g, 22 mmol) was added dropwise. The reaction mixture was stirred at room temperature for 3 h. After the reaction was complete, the precipitated solid was filtered, washed with distilled water (100 mL) and 80% EtOH (100 mL) to give yellow green solid **2**. Intermediate **2** (5.6 g, 16 mmol) was then reacted with 40% glyoxal (3 mL) in a stirred solvent mixture of CH_3_CN (160 mL) and HOAc (40 mL), which was refluxed for 6 h to prepare compound **3**. The solvent was evaporated under vacuum, and 2 N HCl/CH_3_OH (100 mL) was added to the residue. The reaction mixture was stirred at room temperature for 24 h, then evaporated under vacuum and recrystallized from 95% EtOH to obtain scarlet solid **4**; Yield: 62%, m.p.: 180–182 °C. ^1^H- NMR (DMSO-*d*_6_) δ 10.15 (s, 1H), 8.87 (d, *J* = 9.2 Hz, 1H), 8.83 (d, *J* = 9.2 Hz, 1H), 8.40 (d, *J* = 9.2 Hz, 1H), 8.27 (d, *J* = 9.2 Hz, 1H), 7.70 (s, 1H), 5.26 (t, *J* = 6.8 Hz, 2H), 4.15 (s, 3H), 4.09 (s, 3H), 4.03 (s, 3H), 3.94 (s, 3H), 3.65 (t, *J* = 6.8 Hz, 2H). ESI-MS m/z: 376.15. Then, **4** (3.7 g, 9.1 mmol) was heated at 195–210 °C in a dry oven under vacuum (20–30 mmHg) for 40 min. After completion of the reaction, the crude material **5** was obtained, which was used without further purification. Next **5** was acidified with concentrated HCl/EtOH (5:95 by vol.) for 10 min. Then the mixture was evaporated under vacuum and purified with flash column chromatography on silica gel using a CH_2_Cl_2_ and CH_3_OH gradient as eluent to obtain the red solid compound **6**; yield: 90%; m.p.: 176–178 °C; ^1^H-NMR (DMSO-*d_6_*) *δ* 10.07 (s, 1H), 8.78 (d, *J* = 9.6 Hz, 1H), 8.40–8.32 (m, 2H), 8.06 (d, *J* = 8.4 Hz, 1H), 7.67 (s, 1H), 5.18 (t, *J* = 6.6 Hz, 2H), 4.04 (s, 6H), 3.95 (s, 3H), 3.62 (t, *J* = 6.6 Hz, 2H); ^13^C-NMR (DMSO-*d_6_*) *δ* 151.0, 146.9, 146.1, 141.3, 128.4, 128.0 (2), 127.4, 127.3, 123.8, 123.7, 121.8, 121.0, 116.0, 115.8, 114.5, 112.6, 61.2, 56.8, 56.6, 54.9, 26.3; HRMS: calcd for C_22_H_20_NO_4_Cl [M − Cl]^+^: 362.1387, found: 362.1390.

#### 3.2.2. Synthesis of 1,2,8-Trihydroxy-9-methoxycycloberberine Chloride (**10**)

CBBR (3.95 g, 10 mmol) and phloroglucin (5.0 g, 40 mmol) were added into 60% H_2_SO_4_ (20 mL), and the reaction mixture was stirred at 90 °C for 10 h. Then the mixture was cooled, poured into saturated sodium chloride solution (10 mL) and filtered. The residue was washed with water and redissolved in 1 N NaOH (60 mL). After that, 2 N HCl (60 mL) was added and the mixture filtered. The residue was purified with flash column chromatography on silica gel using CH_2_Cl_2_ and CH_3_OH to obtain the intermediate **8** in 62% yield. Next **8** was heated at 195–210 °C in a dry oven under vacuum (20–30 mmHg) for 40 min to create the crude material **9**, which was acidified in concentrated HCl/EtOH (5:95 by vol.) for 10 min. Then the mixture was filtered and the residue was washed with water, CH_2_Cl_2_ and EtOH to afford the title compound **10** as a black-purple solid; yield: 39%; m.p: 191–193 °C; ^1^H-NMR (DMSO-*d_6_*) *δ* 10.30 (s, 1H), 10.00 (s, 1H), 9.55 (s, 1H), 8.67 (d, *J* = 9.6 Hz, 1H), 8.44–8.35 (m, 2H), 8.08 (d, *J* = 9.0 Hz, 1H), 7.29 (s, 1H), 5.11 (t, *J* = 6.6 Hz, 2H), 4.03 (s, 3H), 3.48 (t, *J* = 6.6 Hz, 2H); ^13^C-NMR (DMSO-*d_6_*) *δ* 146.2, 145.5, 144.1, 137.9, 128.6, 127.6, 124.2, 123.9 (3), 122.1, 121.2, 118.8, 117.3, 115.7, 114.8, 56.7, 55.9, 55.2, 25.6; HRMS: calcd for C_20_H_16_NO_4_Cl [M − Cl]^+^: 334.1074, found: 334.1079.

#### 3.2.3. General Procedure for the Synthesis of **13a**–**t**

To a stirred solution of compound **11** (361 mg, 1.0 mmol, 1.0 equiv) and K_2_CO_3_ (2.0 equiv) in anhydrous CH_3_CN (20 mL) were added various benzyl bromides (15.0 equiv). The reaction mixture was refluxed for 3–24 h, cooled, the mixture was filtered and the filtrate was purified by flash column chromatography on silica gel using a gradient of CH_2_Cl_2_ and CH_3_OH as eluent to obtain the compounds **12a**–**t**. Then **12a**–**t** were heated at 195–210 °C in a dry oven under vacuum (20–30 mmHg) for 40 min to create the crude materials, which were acidified in concentrated HCl/EtOH (5:95 by vol.) for 10 min. Then the mixture was evaporated under vacuum and purified by flash column chromatography on silica gel using a CH_2_Cl_2_ and CH_3_OH gradient as eluent to obtain the desired compounds **13a**–**t**.

*1,2-Methylenedioxy-8-hydroxy-9-methoxy-13-benzylcycloberberine chloride* (**13a**): red solid; yield: 78%; m.p.: 248–250 °C; ^1^H-NMR (CDCl_3_/CD_3_OD) *δ* 9.91 (s, 1H), 8.20 (s, 1H), 8.11 (d, *J* = 9.0 Hz, 1H), 7.99 (d, *J* = 9.0 Hz, 1H), 7.39 (s, 1H), 7.36–7.31 (m, 2H), 7.29–7.24 (m, 3H), 6.22 (s, 2H), 5.14 (t, *J* = 6.6 Hz, 2H), 4.78 (s, 2H), 4.11 (s, 3H), 3.66 (t, *J* = 6.6 Hz, 2H); ^13^C-NMR (CDCl_3_/CD_3_OD) *δ* 148.6, 147.2, 146.7, 143.3, 140.5, 140.0, 129.6 (2), 129.2 (2), 128.8, 128.3, 127.1 (2), 126.8, 124.7, 123.6, 121.5, 119.4, 118.1, 116.8, 114.2, 111.3, 102.9, 57.2, 56.7, 41.1, 28.1; HRMS: calcd for C_28_H_22_NO_4_Cl [M – Cl]^+^: 436.1543, found: 436.1535.

*1,2-Methylenedioxy-8-hydroxy-9-methoxy-13-p-fluorobenzylcycloberberine chloride* (**13b**): red solid; yield: 83%; m.p.: 203–205 °C; ^1^H-NMR (CDCl_3_/CD_3_OD) *δ* 9.93 (s, 1H), 8.33 (s, 1H), 8.22 (d, *J* = 9.0 Hz, 1H), 8.04 (d, *J* = 9.0 Hz, 1H), 7.41 (s, 1H), 7.30–7.24 (m, 2H), 7.04–6.98 (m, 2H), 6.19 (s, 2H), 5.12 (t, *J* = 6.6 Hz, 2H), 4.74 (s, 2H), 4.11 (s, 3H), 3.64 (t, *J* = 6.7 Hz, 2H); ^13^C-NMR (CDCl_3_/CD_3_OD) *δ* 162.8, 149.0, 147.6, 147.2, 146.7, 143.3, 140.3, 136.9, 131.5, 131.4, 129.4, 128.7, 127.9, 125.1, 123.9, 122.2, 119.5, 118.7, 117.2, 116.1, 116.0, 114.7, 111.6, 103.4, 57.4, 57.0, 40.5, 28.3; HRMS: calcd for C_28_H_21_NO_4_FCl [M − Cl]^+^: 454.1449, found: 454.1447.

*1,2-Methylenedioxy-8-hydroxy-9-methoxy-13-2′,4′-difluorobenzylcycloberberine chloride* (**13c**): red solid; yield: 78%; m.p.: 218–220 °C; ^1^H-NMR (DMSO-*d_6_*) *δ* 11.56 (s, 1H), 10.09 (s, 1H), 8.54 (s, 1H), 8.29 (d, *J* = 9.0 Hz, 1H), 8.15 (d, *J* = 9.0 Hz, 1H), 7.56 (s, 1H), 7.30–7.26 (m, 1H), 7.00–6.93 (m, 2H), 6.19 (s, 2H), 5.17 (t, *J* = 6.6 Hz, 2H), 4.67 (s, 2H), 4.07 (s, 3H), 3.59 (t, *J* = 6.6 Hz, 2H); ^13^C-NMR (DMSO-*d_6_*) *δ* 161.7, 160.1, 147.3, 147.0, 146.3, 146.2, 141.7, 136.0, 131.2, 128.6, 127.7, 127.4, 124.9, 123.7, 122.4, 122.3, 118.1, 117.6, 116.3, 114.0, 111.8, 111.0, 104.2, 102.5, 57.4, 55.4, 32.7, 26.9; HRMS: calcd for C_28_H_20_NO_4_F_2_Cl [M − Cl]^+^: 472.1355, found: 472.1346.

*1,2-Methylenedioxy-8-hydroxy-9-methoxy-13-2′,6′-difluorobenzylcycloberberine chloride* (**13d**): red solid; yield: 73%; m.p.: 214–216 °C; ^1^H-NMR (DMSO-*d_6_*) *δ* 11.57 (s, 1H), 10.07 (s, 1H), 8.11 (d, *J* = 9.0 Hz, 1H), 7.90 (s, 1H), 7.84 (d, *J* = 9.0 Hz, 1H), 7.60 (s, 1H), 7.56–7.50 (m, 1H), 7.27–7.20 (m, 2H), 6.33 (s, 2H), 5.15 (t, *J* = 6.6 Hz, 2H), 4.77 (s, 2H), 4.02 (s, 3H), 3.59 (t, *J* = 6.6 Hz, 2H); ^13^C-NMR (DMSO-*d_6_*) *δ* 161.5 (2), 147.5, 147.0, 146.5, 146.2, 142.1, 136.3, 130.3, 128.5, 127.7, 127.1, 125.0, 121.8, 118.7, 117.9, 117.4, 116.3, 114.3, 113.1, 112.4, 112.3, 111.1, 102.6, 57.3, 55.4, 27.6, 26.9; HRMS: calcd for C_28_H_20_NO_4_F_2_Cl [M − Cl]^+^: 472.1355, found: 472.1342.

*1,2-Methylenedioxy-8-hydroxy-9-methoxy-13-p-chlorobenzylcycloberberine chloride* (**13e**): red solid; yield: 88%; m.p.: 199–201 °C; ^1^H-NMR (CDCl_3_/CD_3_OD) *δ* 9.93 (s, 1H), 8.27 (s, 1H), 8.19 (d, *J* = 8.4 Hz, 1H), 8.02 (d, *J* = 8.4 Hz, 1H), 7.39 (s, 1H), 7.29 (d, *J* = 8.4 Hz, 2H), 7.22 (d, *J* = 8.4 Hz, 2H), 6.21 (s, 2H), 5.14 (t, *J* = 6.6 Hz, 2H), 4.75 (s, 2H), 4.12 (s, 3H), 3.67 (t, *J* = 6.6 Hz, 2H); ^13^C-NMR (CDCl_3_/CD_3_OD) *δ* 148.6, 147.3, 146.8, 146.1, 143.1, 139.7, 138.9, 132.9, 131.0 (2), 129.2 (2), 128.9, 128.3, 127.0, 124.9, 123.6, 121.9, 119.2, 118.2, 116.8, 114.4, 111.4, 103.0, 57.3, 56.8, 40.5, 28.1; HRMS: calcd for C_28_H_21_NO_4_Cl_2_ [M − Cl]^+^: 470.1154, found: 470.1144.

*1,2-Methylenedioxy-8-hydroxy-9-methoxy-13-p-bromobenzylcycloberberine chloride* (**13f**): red solid; yield: 88%; m.p.: 207–209 °C; ^1^H-NMR (CDCl_3_/CD_3_OD) *δ* 9.93 (s, 1H), 8.22 (s, 1H), 8.15 (d, *J* = 8.4 Hz, 1H), 7.99 (d, *J* = 8.4 Hz, 1H), 7.44 (d, *J* = 7.8 Hz, 2H), 7.38 (s, 1H), 7.15 (d, *J* = 8.4 Hz, 2H), 6.21 (s, 2H), 5.15 (t, *J* = 6.6 Hz, 2H), 4.73 (s, 2H), 4.12 (s, 3H), 3.68 (t, *J* = 6.6 Hz, 2H); ^13^C-NMR (CDCl_3_/CD_3_OD) *δ* 148.4, 147.1, 146.6, 145.7, 142.9, 139.4, 139.0, 132.1 (2), 131.1 (2), 128.6, 128.0, 126.6, 124.6, 123.4, 121.6, 120.8, 119.0, 117.9, 116.5, 114.1, 111.3, 102.8, 57.2, 56.6, 40.4, 28.0; HRMS: calcd for C_28_H_21_BrNO_4_Cl [M − Cl]^+^: 514.0648, found: 514.0653.

*1,2-Methylenedioxy-8-hydroxy-9-methoxy-13-o-methylbenzylcycloberberine chloride* (**13g**): red solid; yield: 83%; m.p.: 203–205 °C; ^1^H-NMR (DMSO-*d_6_*) *δ* 11.54 (s, 1H), 10.09 (s, 1H), 8.34 (s, 1H), 8.15 (s, 2H), 7.57 (s, 1H), 7.29 (d, *J* = 7.8 Hz, 1H), 7.16 (t, *J* = 7.2 Hz, 1H), 7.05 (t, *J* = 7.2 Hz, 1H), 6.73 (d, *J* = 7.5 Hz, 1H), 6.16 (s, 2H), 5.18 (t, *J* = 6.6 Hz, 2H), 4.66 (s, 2H), 4.06 (s, 3H), 3.34 (s, 2H), 2.38 (s, 3H); ^13^C-NMR (DMSO-*d_6_*) *δ* 146.8, 146.2, 145.8, 145.7, 141.4, 138.2, 137.2, 135.8, 129.9, 129.5, 127.9, 127.7, 126.9, 126.1, 125.9, 124.4, 121.8, 121.1, 118.1, 117.1, 115.8, 113.2, 110.4, 101.9, 56.8, 54.9, 37.1, 26.4, 19.2; HRMS: calcd for C_29_H_24_NO_4_Cl [M − Cl]^+^: 450.1700, found: 450.1687.

*1,2-Methylenedioxy-8-hydroxy-9-methoxy-13-m-methylbenzylcycloberberine chloride* (**13h**): red solid; yield: 79%; m.p.: 203–205 °C; ^1^H-NMR (DMSO-*d_6_*) *δ* 11.52 (s, 1H), 10.07 (s, 1H), 8.68 (s, 1H), 8.44 (d, *J* = 9.0 Hz, 1H), 8.17 (d, *J* = 9.0 Hz, 1H), 7.55 (s, 1H), 7.20–7.10 (m, 2H), 7.06 (d, *J* = 7.8 Hz, 1H), 6.99 (d, *J* = 7.2 Hz, 1H), 6.27 (s, 2H), 5.15 (t, *J* = 6.6 Hz, 2H), 4.68 (s, 2H), 4.08 (s, 3H), 3.58 (t, *J* = 6.6 Hz, 2H), 2.23 (s, 3H); ^13^C-NMR (DMSO-*d_6_*) *δ* 146.7, 146.2, 145.7, 145.7, 141.2, 140.0, 137.8, 137.3, 129.5, 129.0, 128.2, 127.8, 127.0, 126.7, 125.5, 124.3, 122.0, 121.8, 117.7, 117.1, 115.8, 113.7, 110.4, 101.8, 56.9, 54.9, 39.7, 26.4, 21.0; HRMS: calcd for C_29_H_24_NO_4_Cl [M − Cl]^+^: 450.1700, found: 450.1687.

*1,2-Methylenedioxy-8-hydroxy-9-methoxy-13-p-methylbenzylcycloberberine chloride* (**13i**): red solid; yield: 81%; m.p.: 229–231 °C; ^1^H-NMR (DMSO-*d_6_*) *δ* 11.51 (s, 1H), 10.06 (s, 1H), 8.67 (s, 1H), 8.43 (d, *J* = 9.0 Hz, 1H), 8.16 (d, *J* = 9.0 Hz, 1H), 7.55 (s, 1H), 7.17 (d, *J* = 7.8 Hz, 2H), 7.07 (d, *J* = 8.4 Hz, 2H), 6.26 (s, 2H), 5.15 (t, *J* = 6.6 Hz, 2H), 4.66 (s, 2H), 4.08 (s, 3H), 3.57 (t, *J* = 6.6 Hz, 2H), 2.22 (s, 3H); ^13^C-NMR (DMSO-*d_6_*) *δ* 147.3, 146.7, 146.2, 146.2, 141.7, 138.6, 137.6, 135.5, 129.4 (2), 128.8 (2), 128.4, 127.6, 127.6, 124.8, 122.5, 122.2, 118.2, 117.7, 116.3, 114.3, 110.9, 102.4, 57.4, 55.4, 39.3, 27.0, 21.0; HRMS: calcd for C_29_H_24_NO_4_Cl [M − Cl]^+^: 450.1700, found: 450.1704.

*1,2-Methylenedioxy-8-hydroxy-9-methoxy-13-3*′,*5*′*-dimethylbenzylcycloberber ine chloride* (**13j**): red solid; yield: 83%; m.p.: 218–220 °C; ^1^H-NMR (DMSO-*d_6_*) *δ* 11.48 (s, 1H), 10.03 (s, 1H), 8.64 (s, 1H), 8.42 (d, *J* = 9.0 Hz, 1H), 8.15 (d, *J* = 9.0 Hz, 1H), 7.53 (s, 1H), 6.87 (s, 2H), 6.78 (s, 1H), 6.25 (s, 2H), 5.12 (t, *J* = 6.6 Hz, 2H), 4.61 (s, 2H), 4.06 (s, 3H), 3.55 (t, *J* = 6.6 Hz, 2H), 2.15 (s, 6H); ^13^C-NMR (DMSO-*d_6_*) *δ* 146.7, 146.1, 145.7 (2), 141.2, 139.9, 137.9, 137.1, 129.5, 127.8, 127.5, 127.1, 127.0, 126.2 (2), 124.2, 122.0, 121.8, 117.7, 117.1, 115.8, 113.8, 110.4, 101.8, 56.9, 54.9, 39.5, 26.4, 20.9 (2); HRMS: calcd for C_30_H_26_NO_4_Cl [M − Cl]^+^: 464.1856, found: 464.1848.

*1,2-Methylenedioxy-8-hydroxy-9-methoxy-13-p-tert-butylmethylbenzylcyclo berberine chloride* (**13k**): red solid; yield: 77%; m.p.: 212–214 °C; ^1^H-NMR (DMSO-*d_6_*) *δ* 11.51 (s, 1H), 10.06 (s, 1H), 8.73 (s, 1H), 8.48 (d, *J* = 9.0 Hz, 1H), 8.17 (d, *J* = 9.0 Hz, 1H), 7.56 (s, 1H), 7.30–7.21 (m, 4H), 6.29 (s, 2H), 5.14 (t, *J* = 6.6 Hz, 2H), 4.68 (s, 2H), 4.09 (s, 3H), 3.54 (t, *J* = 6.6 Hz, 2H), 1.21 (s, 9H); ^13^C-NMR (DMSO-*d_6_*) *δ* 148.3, 146.7, 146.1, 145.7, 141.1, 138.1, 137.2, 132.5, 128.0 (2), 127.8, 127.2, 127.0, 125.0 (2), 124.2, 122.0, 121.8, 117.6, 117.2, 115.8, 113.8, 110.3, 101.8, 56.9, 54.9, 48.5, 38.5, 31.0 (3), 26.4; HRMS: calcd for C_32_H_30_NO_4_Cl [M − Cl]^+^: 492.2169, found: 492.2158.

*1,2-Methylenedioxy-8-hydroxy-9-methoxy-13-p-trifluoromethoxybenzylcyclo berberine chloride* (**13l**): red solid; yield: 77%; m.p.: 185–187 °C; ^1^H-NMR (DMSO-*d_6_*) *δ* 11.53 (s, 1H), 10.08 (s, 1H), 8.78 (s, 1H), 8.48 (d, *J* = 9.0 Hz, 1H), 8.17 (d, *J* = 9.0 Hz, 1H), 7.56 (s, 1H), 7.42 (d, *J* = 7.8 Hz, 2H), 7.26 (d, *J* = 8.4 Hz, 2H), 6.26 (s, 2H), 5.15 (t, *J* = 6.6 Hz, 2H), 4.73 (s, 2H), 4.09 (s, 3H), 3.58 (t, *J* = 6.6 Hz, 2H); ^13^C-NMR (DMSO-*d_6_*) *δ* 146.8, 146.6, 146.3, 145.7 (2), 141.0, 139.8, 137.1, 130.1 (2), 129.5, 127.9, 127.2, 127.0, 124.3, 122.2, 122.0, 120.9 (2), 117.4, 117.2, 115.8, 113.8, 110.4, 101.9, 56.9, 54.9, 38.4, 26.4; HRMS: calcd for C_29_H_21_NO_5_F_3_Cl [M − Cl]^+^: 520.1366, found: 520.1349.

*1,2-Methylenedioxy-8-hydroxy-9-methoxy-13-o-cyanbenzylcycloberberine chloride* (**13m**): red solid; yield: 83%; m.p.: 209–211 °C; ^1^H-NMR (CDCl_3_/CD_3_OD) *δ* 9.96 (s, 1H), 8.27 (s, 1H), 8.14 (d, *J* = 9.0 Hz, 1H), 8.01 (d, *J* = 9.0 Hz, 1H), 7.80 (dd, *J* = 7.8, 1.2 Hz, 1H), 7.54 (td, *J* = 7.8, 1.2 Hz, 1H), 7.44 (t, *J* = 7.8 Hz, 1H), 7.40 (s, 1H), 7.19 (d, *J* = 7.8 Hz, 1H), 6.19 (s, 2H), 5.16 (t, *J* = 6.6 Hz, 2H), 4.98 (s, 2H), 4.12 (s, 3H), 3.68 (t, *J* = 6.6 Hz, 2H); ^13^C-NMR (CDCl_3_/CD_3_OD) *δ* 148.6, 147.4, 146.8, 146.3, 144.0, 142.9, 137.5, 133.9, 133.8, 130.1, 129.0, 128.2, 128.0, 127.0, 124.9, 123.4, 122.2, 118.9, 118.6, 118.1, 116.7, 114.2, 113.2, 111.5, 103.0, 57.2, 56.7, 39.5, 28.0; HRMS: calcd for C_29_H_21_N_2_O_4_Cl [M − Cl]^+^: 461.1496, found: 461.1502.

*1,2-Methylenedioxy-8-hydroxy-9-dimethoxy-13-m-cyanbenzylcycloberberine chloride* (**13n**): red solid; yield: 87%; m.p.: 189–191 °C; ^1^H-NMR (CD_3_OD) *δ* 9.95 (s, 1H), 8.50 (s, 1H), 8.33 (d, *J* = 9.0 Hz, 1H), 8.07 (d, *J* = 9.0 Hz, 1H), 7.58 (td, *J* = 9.0, 1.2 Hz, 2H), 7.55 (s, 1H), 7.45 (t, *J* = 7.8 Hz, 1H), 7.42 (s, 1H), 6.15 (s, 2H), 5.13 (t, *J* = 6.6 Hz, 2H), 4.81 (s, 2H), 4.11 (s, 3H), 3.64 (t, *J* = 6.6 Hz, 2H); ^13^C-NMR (CD_3_OD) *δ* 151.0, 148.4, 146.9, 145.8, 142.5, 141.3, 138.2, 133.8, 132.3, 130.6, 129.8, 129.2, 128.5, 126.9, 126.3, 123.2, 121.7, 120.7, 119.0, 118.7, 118.5, 117.5, 112.6, 111.4, 102.5, 57.2, 56.8, 40.3, 27.6; HRMS: calcd for C_29_H_21_N_2_O_4_Cl [M − Cl]^+^: 461.1496, found: 461.1495.

*1,2-Methylenedioxy-8-hydroxy-9-dimethoxy-13-p-cyanbenzylcycloberberine chloride* (**13o**): red solid; yield: 83%; m.p.:197–199 °C; ^1^H-NMR (DMSO-*d_6_*) *δ* 11.54 (s, 1H), 10.09 (s, 1H), 8.78 (s, 1H), 8.46 (d, *J* = 9.0 Hz, 1H), 8.16 (d, *J* = 9.0 Hz, 1H), 7.74 (d, *J* = 8.4 Hz, 2H), 7.55 (s, 1H), 7.46 (d, *J* = 7.8 Hz, 2H), 6.21 (s, 2H), 5.16 (t, *J* = 6.6 Hz, 2H), 4.78 (s, 2H), 4.09 (s, 3H), 3.58 (t, *J* = 6.6 Hz, 2H); ^13^C-NMR (DMSO-*d_6_*) *δ* 146.8, 146.4, 146.2, 145.8, 145.7, 140.9, 136.0, 132.2 (2), 129.31 (2), 128.0, 127.2, 127.0, 124.3, 122.5, 121.9, 118.8, 117.4, 117.1, 115.8, 113.8, 110.5, 108.9, 101.9, 56.9, 54.9, 39.2, 26.4; HRMS: calcd for C_29_H_21_N_2_O_4_Cl [M − Cl]^+^: 461.1496, found: 461.1500.

*1,2-Methylenedioxy-8-hydroxy-9-methoxy-13-o-nitrobenzylcycloberberine chloride* (**13p**): red solid; yield: 82%; m.p.: 199–201 °C; ^1^H-NMR (DMSO-*d_6_*) *δ* 11.57 (s, 1H), 10.11 (s, 1H), 8.69 (s, 1H), 8.35 (d, *J* = 9.0 Hz, 1H), 8.18 (d, *J* = 9.0 Hz, 1H), 8.05 (dd, *J* = 7.8, 1.2 Hz, 1H), 7.56–7.46 (m, 3H), 7.07 (dd, *J* = 7.8, 1.2 Hz, 1H), 6.04 (s, 2H), 5.17 (t, *J* = 6.6 Hz, 2H), 4.88 (s, 2H), 4.08 (s, 3H), 3.58 (t, *J* = 6.6 Hz, 2H); ^13^C-NMR (DMSO-*d_6_*) *δ* 149.7, 147.3, 147.1, 146.4, 146.3, 141.4, 135.9, 134.8, 133.7, 131.0, 130.1, 128.6, 128.1, 127.8, 127.5, 124.9, 123.1, 122.3, 118.0, 117.6, 116.4, 114.2, 111.0, 102.3, 57.4, 55.4, 36.7, 26.9; HRMS: calcd for C_28_H_21_N_2_O_6_Cl [M − Cl]^+^: 481.1394, found: 481.1381.

*1,2-Methylenedioxy-8-hydroxy-9-methoxy-13-m-nitrobenzylcycloberberine chloride* (**13q**): red solid; yield: 89%; m.p.: 186–188 °C; ^1^H-NMR (CDCl_3_/CD_3_OD) *δ* 9.97 (s, 1H), 8.47 (s, 1H), 8.31 (d, *J* = 8.4 Hz, 1H), 8.12–8.08 (m, 2H), 8.05 (d, *J* = 9.0 Hz, 1H), 7.69 (d, *J* = 7.8 Hz, 1H), 7.54 (t, *J* = 8.4 Hz, 1H), 7.40 (s, 1H), 6.20 (s, 2H), 5.16 (t, *J* = 6.6 Hz, 2H), 4.90 (s, 2H), 4.13 (s, 3H), 3.68 (t, *J* = 6.6 Hz, 2H); ^13^C-NMR (CDCl_3_/CD_3_OD) *δ* 149.2, 148.7, 147.5, 146.9, 146.4, 142.8 (2), 138.2, 135.9, 130.2, 129.2, 128.4, 127.2, 125.0, 124.0, 123.7, 122.7, 122.2, 119.0, 118.3, 116.9, 114.5, 111.6, 103.0, 57.3, 56.8, 40.7, 28.1; HRMS: calcd for C_28_H_21_N_2_O_6_Cl [M − Cl]^+^: 481.1394, found: 481.1392.

*1,2-Methylenedioxy-8-hydroxy-9-methoxy-13-p-nitrobenzylcycloberberine chloride* (**13r**): red solid; yield: 85%; m.p.: 186–188 °C; ^1^H-NMR (CDCl_3_/CD_3_OD) *δ* 9.98 (s, 1H), 8.49 (s, 1H), 8.33 (d, *J* = 9.0 Hz, 1H), 8.16 (s, 1H), 8.15 (s, 1H), 8.07 (d, *J* = 9.0 Hz, 1H), 7.47 (d, *J* = 8.4 Hz, 2H), 7.41 (s, 1H), 6.17 (s, 2H), 5.15 (t, *J* = 6.6 Hz, 2H), 4.91 (s, 2H), 4.13 (s, 3H), 3.66 (t, *J* = 6.6 Hz, 2H); ^13^C-NMR (CDCl_3_/CD_3_OD) *δ* 148.9, 148.8, 147.6 (2), 147.1, 146.7, 143.0, 138.2, 130.4 (2), 129.4, 128.6, 127.6, 125.1, 124.4 (2), 123.8, 123.0, 119.2, 118.5, 117.1, 114.7, 111.7, 103.2, 57.4, 56.9, 41.1, 28.2; HRMS: calcd for C_28_H_21_N_2_O_6_Cl [M − Cl]^+^: 481.1394, found: 481.1390.

*1,2-Methylenedioxy-8-hydroxy-9-methoxy-13-1′-phenylethylcycloberberine chloride* (**13s**): orange solid; yield: 82%; m.p.: 213–215 °C; ^1^H-NMR (DMSO-*d_6_*) *δ* 11.53 (s, 1H), 10.07 (s, 1H), 8.49 (s, 1H), 8.43 (d, *J* = 9.0 Hz, 1H), 8.13 (d, *J* = 9.0 Hz, 1H), 7.56 (s, 1H), 7.41–7.37 (m, 2H), 7.30 (t, *J* = 7.8 Hz, 2H), 7.19 (tt, *J* = 7.2, 1.2 Hz, 1H), 6.35 (s, 1H), 6.23–6.20 (m, 1H), 5.48 (q, *J* = 6.6 Hz, 1H), 5.20–5.06 (m, 2H), 4.08 (s, 3H), 3.57 (t, *J* = 6.6 Hz, 2H), 1.87 (d, *J* = 7.2 Hz, 3H); ^13^C-NMR (DMSO-*d_6_*) *δ* 147.4, 146.8, 146.3, 146.2, 145.7, 144.0, 141.6, 128.8 (2), 128.3, 128.2 (2), 127.8, 127.5, 126.7, 124.7, 122.2, 119.0, 118.2, 117.7, 116.3, 114.3, 111.0, 102.2, 57.4, 55.4, 41.0, 27.0, 22.7; HRMS: calcd for C_29_H_24_NO_4_Cl [M − Cl]^+^: 450.1700, found: 450.1706.

*1,2-Methylenedioxy-8-hydroxy-9-methoxy-13-2′-methoxy-2′-oxo-1′-phenylethylcycloberberine chloride* (**13t**): orange solid; yield: 84%; m.p.: 173–175 °C; ^1^H-NMR (DMSO-*d_6_*) *δ* 11.62 (s, 1H), 10.10 (s, 1H), 8.14 (d, *J* = 9.0 Hz, 1H), 7.97 (s, 1H), 7.76 (s, 1H), 7.61 (s, 1H), 7.53–7.41 (m, 5H), 6.35 (s, 1H), 6.32 (s, 1H), 6.24 (s, 1H), 5.20–5.09 (m, 2H), 4.04 (s, 3H), 3.73 (s, 3H), 3.63–3.57 (m, 2H); ^13^C-NMR (DMSO-*d_6_*) *δ* 171.9, 147.0, 146.9, 146.0, 145.7, 141.0, 137.0, 136.2, 129.5, 129.1 (2), 128.9 (2), 128.1, 127.7, 127.4, 126.7, 124.6, 120.9, 117.6, 117.0, 115.8, 112.3, 110.6, 102.1, 56.8, 54.8, 54.1, 52.4, 26.4; HRMS: calcd for C_30_H_24_NO_6_Cl [M − Cl]^+^: 494.1598, found: 494.1591.

#### 3.2.4. General Procedure for the Synthesis of **15a**–**c**

To a stirred solution of intermediate **11** [15] (361 mg, 1.0 mmol, 1.0 equiv) in 80% EtOH (20 mL), various aliphatic aldehydes (5.0 equiv) and HOAc (4 mL) were added. The reaction mixture was heated to reflux for 8 h. The solvent was removed by evaporation to afford a red oil. The red oil was acidified by 2% HCl, stirred at room temperature for 0.5 h. The solvent was removed and the residue was purified by flash chromatography over silica gel using CH_2_Cl_2_/CH_3_OH (5:1) as eluent to obtain the intermediate compounds **14a–c**. Next **14a–c** were heated at 195–210 ^o^C in a dry oven under vacuum (20–30 mmHg) for 40 min to create the crude materials, to were acidified in concentrated HCl/EtOH (5:95 by vol.) for 10 min. Then the mixture was evaporated under vacuum and purified by flash column chromatography on silica gel using a gradient of CH_2_Cl_2_ and CH_3_OH as eluent to obtain the desired compounds **15a***–***c**.

*1,2-Methylenedioxy-8-hydroxy-9-methoxy-13-ethylcycloberberine chloride* (**15a**): orange solid; yield: 82%; m.p.: 166–168 °C; ^1^H-NMR (DMSO-*d_6_*) *δ* 11.49 (s, 1H), 10.05 (s, 1H), 8.54 (s, 1H), 8.49 (d, *J* = 9.0 Hz, 1H), 8.12 (d, *J* = 9.0 Hz, 1H), 7.55 (s, 1H), 6.34 (s, 2H), 5.14 (t, *J* = 6.6 Hz, 2H), 4.08 (s, 3H), 3.57 (t, *J* = 6.6 Hz, 2H), 3.30 (q, *J* = 7.2 Hz, 2H), 1.36 (t, *J* = 7.2 Hz, 3H); ^13^C-NMR (DMSO-*d_6_*) *δ* 146.8, 145.9, 145.7 (2), 141.5, 141.2, 127.6, 127.1 (2), 124.2, 122.2, 119.8, 117.5, 117.1, 115.9, 114.1, 110.3, 102.0, 57.0, 55.0, 28.0, 26.6, 16.1; HRMS: calcd for C_23_H_20_NO_4_Cl [M − Cl]^+^: 374.1387, found: 374.1396.

*1,2-Methylenedioxy-8-hydroxy-9-methoxy-13-propylcycloberberine chloride* (**15b**): orange solid; yield: 85%; m.p.: 165–167 °C; ^1^H-NMR (DMSO-*d_6_*) *δ* 11.49 (s, 1H), 10.04 (s, 1H), 8.58 (s, 1H), 8.53 (d, *J* = 9.0 Hz, 1H), 8.15 (d, *J* = 9.0 Hz, 1H), 7.55 (s, 1H), 6.34 (s, 2H), 5.15 (t, *J* = 6.6 Hz, 2H), 4.09 (s, 3H), 3.58 (t, *J* = 6.6 Hz, 2H), 3.28 (t, *J* = 7.8 Hz, 2H), 1.80–1.72 (m, 2H), 1.02 (t, *J* = 7.2 Hz, 3H); ^13^C-NMR (DMSO-*d_6_*) *δ* 146.6, 145.8, 145.6 (2), 141.2, 139.7, 127.6, 127.1, 127.0, 124.1, 122.0, 120.5, 117.6, 117.1, 115.8, 114.0, 110.2, 101.9, 56.9, 54.9, 36.5, 26.4, 24.3, 13.72; HRMS: calcd for C_24_H_22_NO_4_Cl [M − Cl]^+^: 388.1543, found: 388.1553.

*1,2-Methylenedioxy-8-hydroxy-9-methoxy-13-butylcycloberberine chloride* (**15c**): orange solid; yield: 88%; m.p.: 162–164 °C; ^1^H-NMR (DMSO-*d_6_*) *δ* 11.45 (s, 1H), 10.02 (s, 1H), 8.55 (s, 1H), 8.50 (d, *J* = 9.0 Hz, 1H), 8.12 (d, *J* = 9.0 Hz, 1H), 7.53 (s, 1H), 6.31 (s, 2H), 5.12 (t, *J* = 6.6 Hz, 2H), 4.06 (s, 3H), 3.55 (t, *J* = 6.6 Hz, 2H), 3.27 (t, *J* = 7.8 Hz, 2H), 1.68 (t, *J* = 7.8 Hz, 2H), 1.46–1.38 (m, 2H), 0.93 (t, *J* = 7.2 Hz, 3H); ^13^C-NMR (DMSO-*d_6_*) *δ* 146.7, 145.8, 145.6 (2), 141.2, 140.0, 127.6, 127.1, 127.0, 124.1, 122.1, 120.4, 117.6, 117.1, 115.8, 114.0, 110.2, 101.9, 56.9, 54.9, 34.3, 33.3, 25.4, 22.0, 13.7; HRMS: calcd for C_25_H_24_NO_4_Cl [M − Cl]^+^: 402.1700, found: 402.1692.

### 3.3. Antimicrobial Assay

Minimum inhibitory concentrations (MICs) of the target compounds were determined by using the agar dilution assay at various concentrations of 64, 32, 16, 8.0, 4.0, 2.0, 1.0, 0.5, 0.25, 0.125, 0.06 and 0.03 mg/mL described by the Clinical Laboratory Standards Institute [17]. Bacterial strains were purchased from the ATCC collection or isolated from Chinese hospitals. The medium was Mueller-Hinton agar, and the inoculum was 10^4^ colony forming units (cfu)/spot. Culture plates were incubated at 35 °C for 18 h, and MICs were defined as the lowest concentrations that prevented visible growth of the bacteria.

### 3.4. Time-kill Curves of **15a**

Kill kinetics of **15a** was determined by time-kill experiments for MRSA strain (clinical isolate MRSA 18-4) according to the method described by Verma et al. with slight modifications [25]. An overnight culture was diluted with CAMH broth in a total volume of 30 mL containing an inoculum of 2×10^6^ CFU/mL in a 250 mL flask for 18-4. Distilled water or **15a** was added to yield concentrations of 0×, 1/2×, 2× and 8× MIC in the broth at standard inocula. Viability counts were performed at 0, 2, 4, 8 and 12 h of incubation at 37 °C by plating 0.1 mL undiluted and 10-fold serial diluted samples onto TSA plates in duplicate. Drug carryover effect was eliminated by saline and agar dilution. The experiments were performed three times on different days and the results were presented as mean and standard deviation.

### 3.5. Stability Assay of Key Compound in Liver Microsomes

The assay was performed with liver microsomes from CD-1 mouse, SD rat and human. Microsomes in 100 mM potassium phosphate buffer (0.5 mg/mL microsomal protein), cofactor MgCl_2_ (10 mM), tested compound (1 μM, cosolvent (0.01% DMSO), 0.99% CH_3_OH), and then NADPH (1 mM) at 37 °C for 60 min. The reaction can be started by the addition of liver microsomes or the tested compound or NADPH. Aliquots were sampled at 0, 5, 10, 20, 30 and 60 min incubation, and enzymatic reaction was stopped by protein precipitation in cold acetonitrile including 100 ng/mL tolbutamide and 100 ng/mL labetalol as internal standard. After centrifugation, samples were then analyzed by LC/MS/MS [26].

### 3.6. Stability Assay of Key Compound in SD Rat Whole Blood

Fresh blood was collected on the day of experiment from SD rats and pre-warmed in a water bath at 37 °C. 10 mM test compound or Ems stock solutions were prepared in DMSO, and then diluted with 45% MeOH/H_2_O to achieve 100 μM dosing solutions. Each dosing solution (2 μL) was incubated with 98 µL of blank blood at 37 °C in water bath for 0, 0.5, 1, 3, 7 and 24 h, respectively. At the end of incubation, for each sample, 100 μL water and 800 μL of stop solution (200 ng/mL tolbutamide plus 20 ng/mL buspirone in ACN) were immediately added to to precipitate protein and centrifuge at 4,000 rpm for 20 min. An aliquot of supernatant (100 μL) was then extracted, mixed with 200 μL H_2_O and then shook at 800 rpm for about 10 min before submitting to LC-MS/MS analysis. The experiment was repeated two times [18].

### 3.7. Acute Toxicity Assay

Female Kunming mice with weight of 20.0 (±1.0 g) were fed with regular rodent chow and housed in an air conditioned room. The mice were randomly divided into different groups with 6 mice each. Each compound was given intravenously in a single-dosing experiment at 40, 60, or 80 mg·kg^−1^ (ddH_2_O as control), respectively. The mice were closely monitored for 7 days. Body weight as well as survival was monitored [18].

### 3.8. Enzymatic Assay

The gyrase supercoling assay kit and topo IV decatenation kit were the tools for detection the target compound inhibited the activity of DNA gyrase and topo IV from MRSA (both kits obtained from Inspiralis, Norwich, United Kingdom). Briefly, supercoiled pBR322 plasmid DNA (0.5 mg) and **13a** of varying concentrations (2.5–100 μg/mL) were incubated with 1 U gyrase in the dedicated supercoiling assay buffer supplied by the manufacturer. Reactions were carried out at 37 °C for 30 min and then terminated by the addition of equal volume of STEB buffer (40% sucrose, 100 mM Tris-HCl pH 7.5, 1 mM EDTA, 0.5 mg/ml bromophenol blue) and chloroform/isoamyl alcohol. Samples were vortexed, centrifuged and run through a 15 cm 1% agarose gel in TAE buffer (40 mM Tris-acetate, 2 mM EDTA) for 3 h at 50 V. Gels were stained with ethidium bromide and visualized under UV light. The decatenation assay was performed using MRSA topo IV decatenation kit. The operational approach was similar to above. Bands were visualized by UV light and photographed [27].

### 3.9. Molecular Docking Assay

The topo IV crystal structure in complex with kibdelomycin (PDB code 4URL, resolution: 2.29 Å) was selected as the template to generate the binding modes [24]. Discovery Studio 4.5 was used to create structures of the ligands and to perform energy minimization [16]. The LibDock software was employed. Before docking, protein structure and ligands were processed [28]. In the course of protein preparation, ligands and water molecules were removed from the structure. The docking conformation with the highest docking score was finally chosen to analyze the receptor ligand interaction in the Discovery Studio 4.5 software.

## 4. Conclusions

A total of 25 new derivatives of **1** were designed, synthesized and evaluated for their anti-Gram-positive microbes activities with **1** as the lead. SAR studies revealed that introducing a small-sized substituent at the 13-position might be beneficial for activity. Part of them showed good activities against MRSA and VISA. Compound **15a** bearing a 13-ethyl group exhibited the most potent antibacterial activities, with MIC values of 0.25–0.5 μg/mL, superior to those of the lead **1**. Time-kill curve analysis of **15a** further confirmed its potential bactericidal activity. Meanwhile, compound **15a** displayed high liver microsomal metabolic stability and reasonable whole blood stability. It also showed a good in vivo safety profile with an LD_50_ value over 65.6 mg·kg^−1^ in mice via the intravenous route. A preliminary mechanism study showed that compound **15a** could inhibit topo IV activity through binding with the active site of kibdelomycin, a different mechanism of action from the quinolone antibiotics currently used in hospitals. Taken together, these results provide powerful information for developing these compounds into promising anti-MRSA candidates with novel chemical scaffolds and a specific biological mechanism of action, and compound **15a** has been chosen for further investigation.

## Figures and Tables

**Table 1:**
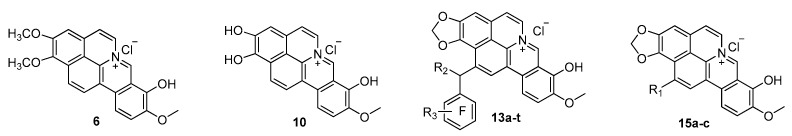
Antimicrobial activities of the target compounds against drug-susceptible Gram-positive strains (MIC (μg/mL) ^a^).

Code	R_1_/R_2_	R_3_	MSSA ^c^				MSSE ^c^			S.s ^c^		S.h ^c^
			ATCC 29213	15 ^b^	16-1 ^b^		ATCC 12228	16-5 ^b^		ATCC 15305		ATCC 35982
CBBR	-	-	>64	>64	>64		>64	>64		>64		>64
**1**	-	-	0.5	4	1		0.5	0.5		>64		1
**6**	-	-	>64	>64	>64		>64	>64		>64		>64
**10**	-	-	32	16	32		8	16		32		32
**13a**	H	H	8	16	8		16	8		8		8
**13b**	H	*p*-F	>64	>64	>64		>64	>64		>64		>64
**13c**	H	2′,4′-(F)_2_	8	8	8		8	8		16		8
**13d**	H	2′,6′-(F)_2_	>64	>64	>64		>64	>64		>64		>64
**13e**	H	*p*-Cl	8	8	4		8	4		16		8
**13f**	H	*p*-Br	4	4	2		4	2		4		4
**13g**	H	*o*-CH_3_	>64	>64	>64		>64	>64		>64		>64
**13h**	H	*m*-CH_3_	4	4	4		4	2		16		8
**13i**	H	*p*-CH_3_	8	8	4		8	4		16		4
**13j**	H	3′,5′-(CH_3_)_2_	32	32	32		32	16		64		32
**13k**	H	*p*-tBu	32	32	16		16	8		32		32
**13l**	H	*p*-OCF_3_	32	32	32		32	32		64		64
**13m**	H	*o*-CN	>64	>64	>64		>64	>64		>64		>64
**13n**	H	*m*-CN	>64	>64	>64		16	32		>64		64
**13o**	H	*p*-CN	8	4	8		8	16		16		16
**13p**	H	*o*-NO_2_	8	8	4		8	4		16		8
**13q**	H	*m*-NO_2_	>64	>64	64		64	32		>64		64
**13r**	H	*p*-NO_2_	16	8	16		4	4		32		8
**13s**	CH_3_	H	16	8	16		32	16		64		32
**13t**	CO_2_CH_3_	H	64	64	>64		64	32		>64		64
**15a**	CH_2_CH_3_	-	0.5	0.5	0.5		0.5	0.25		1		0.5
**15b**	(CH_2_)_2_CH_3_	-	2	2	2		2	1		8		2
**15c**	(CH_2_)_3_CH_3_	-	8	4	4		8	8		16		4
Lev			0.125	0.125	0.25		0.126	0.125		1		0.125

^a^ MIC (μg/mL), minimum inhibitory concentration; ^b^ Strains isolated from patients in China. ^c^ Methicillin-susceptible *S. aureus* (MSSA), methicillin-susceptible *S. epidermidis* (MSSE), *S. saprophyticus* (S. s) and *S. hominis* (S. h).

**Table 2:** Antibacterial activities of the target compounds against drug-resistant Gram-positive strains (MIC (μg/mL)).

Code	MRSA							VISA			MRSE	
	13-18	13-23	16-34	ATCC 43300	ATCC-B AA-1708 ^a^	ATCC -BAA-976 ^b^		ATCC 700698	ATCC 700699		13-3	16-5
CBBR	>64	>64	>64	>64	>64	>64		>64	>64		>64	>64
**1**	1	1	0.5	0.5	0.5	1		1	0.5		>64	0.5
**6**	>64	>64	>64	>64	>64	>64		>64	>64		>64	>64
**10**	32	64	16	32	16	64		32	32		32	16
**13a**	16	16	8	16	8	16		8	8		8	16
**13b**	>64	>64	>64	>64	>64	>64		>64	>64		>64	>64
**13c**	16	8	8	8	4	8		8	8		8	8
**13d**	>64	>64	>64	>64	>64	>64		>64	>64		>64	32
**13e**	8	8	4	8	8	4		4	8		64	4
**13f**	4	4	4	4	1	2		2	1		16	2
**13g**	>64	>64	>64	>64	>64	>64		>64	>64		64	64
**13h**	8	8	4	8	4	8		4	4		4	4
**13i**	8	8	8	16	2	4		4	4		2	2
**13j**	32	32	16	32	16	32		32	32		16	16
**13k**	16	32	16	32	8	16		32	32		8	8
**13l**	32	32	32	64	32	64		64	32		32	32
**13m**	>64	>64	>64	>64	>64	>64		>64	>64		>64	>64
**13n**	>64	>64	>64	>64	>64	>64		>64	>64		64	32
**13o**	8	8	8	4	8	16		4	8		16	8
**13p**	16	8	4	8	4	8		8	4		32	8
**13q**	>64	>64	>64	>64	>64	64		>64	64		64	32
**13r**	32	8	8	64	4	32		4	4		>64	16
**13s**	16	8	16	16	16	32		32	32		32	32
**13t**	64	64	>64	64	64	64		64	64		64	32
**15a**	2	0.5	0.25	0.5	0.25	0.5		0.5	0.25		1	0.25
**15b**	4	4	2	4	2	2		4	2		4	2
**15c**	4	8	8	4	8	4		4	4		16	8
Lev	32	64	64	32	16	0.125		32	8		32	8

^a^ mupA positive (isolates with mupirocin resistance). ^b^ clindamycin-resistant *S. aureus*.

**Table 3:** Metabolic stability of tested compound in human, rat and mouse liver microsomes ^a^.

Code	Species	T_1/2_ (min)	CL Int (mic) (μL/min/mg)	Remaining Ratio (%) (T = 60 min)
**15a**	Human	105.3	13.2	70.6
	Rat	50.9	27.3	47.9
	Mouse	18.9	73.4	10.4
Tes	Human	12.9	107.2	3.9
	Rat	0.6	2349.8	0
	Mouse	5.5	253.9	0

^a^ T_1/2_ = half-life. CL _int (mic)_ = microsomal intrinsic clearance. Liver weight: 20 g/kg for humans.

**Table 4:** Metabolic stability of **15a** in whole blood.

Time (h)	0	0.5	1	3	7	24
**15a** Rr (%)	100	98.9	98.0	95.8	93.1	85.4
Ems Rr (%)	100	37.5	9.1	0	0	0

Rr = Remaining ratio.

## Supplementary Materials

The following are available online at https://www.mdpi.com/1420-3049/24/5/984/s1, the ^1^H-NMR, ^13^C-NMR and HRMS data of compound **15a**.
