# Supplementary material for: Evolution and Antibacterial Evaluation of 8-Hydroxy-cycloberberine Derivatives as a Novel Family of Antibacterial Agents Against MRSA
Source: Molecules. 2019 Mar 11;24(5):984. doi: 10.3390/molecules24050984 (PMC6429263; doi:10.3390/molecules24050984)

Supporting Information

Evolution and antibacterial evaluation of 8-hydroxycycloberberine derivatives as a novel family of bactericidal agents against MRSA

Yuan-Shuai Yang†, Wei Wei†, Xin-Xin Hu, Sheng Tang, Jing Pang, Xue-Fu You, Tian-Yun Fan **, Yan-Xiang Wang * and Dan-Qing Song

Figure S1. ^{1}H NMR spectrum of compound 15a

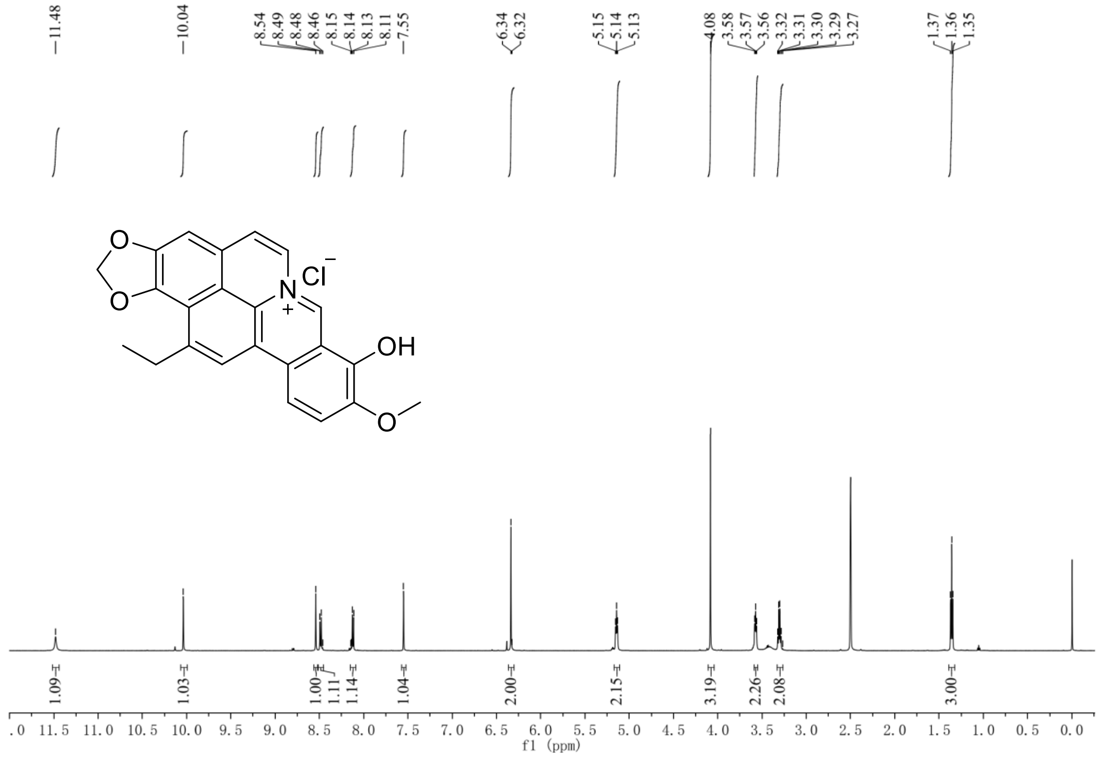

Figure S2. ^{13}C-NMR spectrum of compound 15a

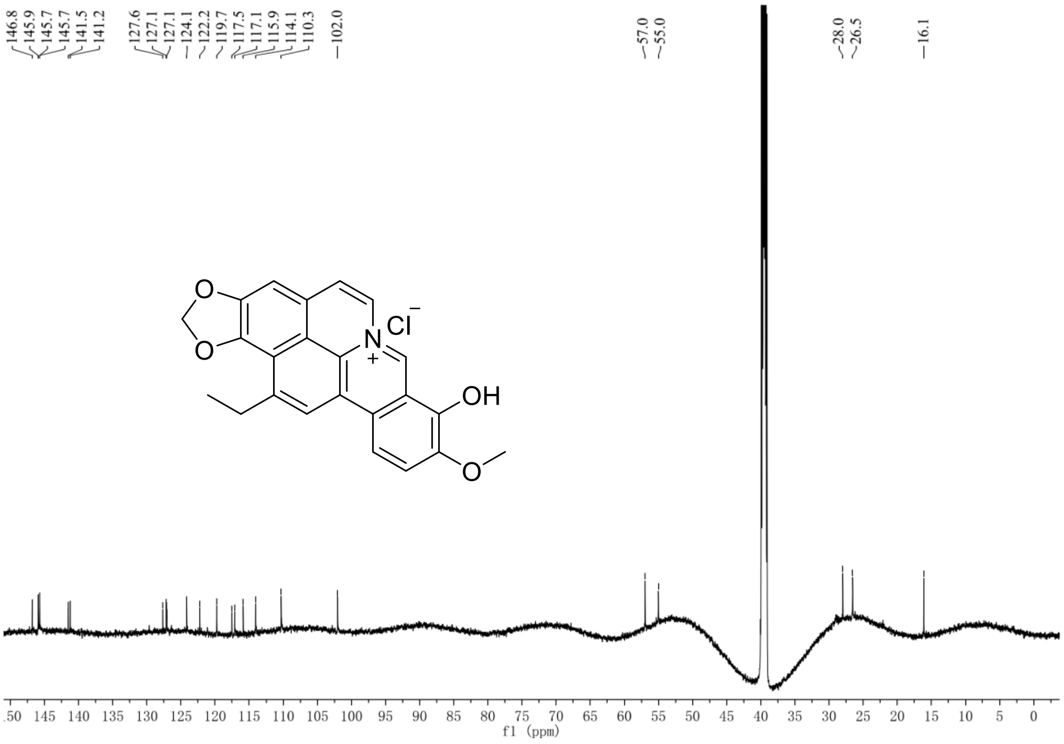

Figure S3. HRMS spectrum of compound 15a

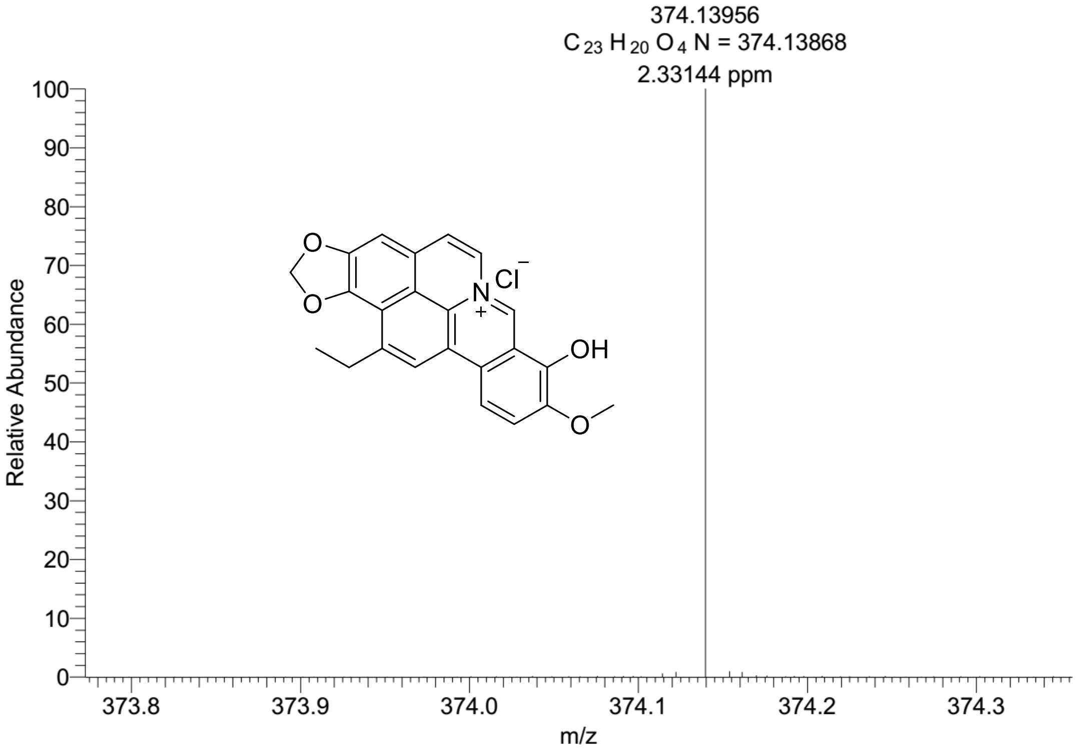

Supplement: Supplementary file 1 [file molecules-24-00984-s001.pdf]
